# Using Anal Cytology and Human Papillomavirus DNA and E6/E7 mRNA Detection to Optimize High-Resolution Anoscopy Referrals in Men Who Have Sex With Men With HIV

**DOI:** 10.1093/ofid/ofae735

**Published:** 2024-12-27

**Authors:** Ana C Silva-Klug, Sònia Paytubi, Montserrat Torres, Loris Trenti, Nuria Baixeras, Monica Sanchez-Llamas, Miquel A Pavon, Silvia De Sanjose, Isabel Catala, August Vidal, Mario Poljak, Laia Alemany, Daniel Podzamczer, Sebastian Videla, Maria Saumoy

**Affiliations:** HIV and STD Unit, Infectious Diseases Department, Bellvitge University Hospital/Bellvitge Biomedical Research Institute, L’Hospitalet de Llobregat, Barcelona, Spain; Infection and Cancer Laboratory, Cancer Epidemiology Research Program, Catalan Institute of Oncology/Bellvitge Biomedical Research Institute, L’Hospitalet de Llobregat, Barcelona, Spain; Centro de investigación Biomédica en Red en Epidemiología y Salud Pública, Ministerio de Ciencia e Innovación en Epidemiología y Salud Pública, Madrid, Spain; Infection and Cancer Laboratory, Cancer Epidemiology Research Program, Catalan Institute of Oncology/Bellvitge Biomedical Research Institute, L’Hospitalet de Llobregat, Barcelona, Spain; Centro de investigación Biomédica en Red en Epidemiología y Salud Pública, Ministerio de Ciencia e Innovación en Epidemiología y Salud Pública, Madrid, Spain; Colorectal Unit, General and Digestive Surgery Department, Bellvitge University Hospital/Bellvitge Biomedical Research Institute, L’Hospitalet de Llobregat, Barcelona, Spain; Pathology Unit, Bellvitge University Hospital/Bellvitge Biomedical Research Institute, L’Hospitalet de Llobregat, Barcelona, Spain; HIV and STD Unit, Infectious Diseases Department, Bellvitge University Hospital/Bellvitge Biomedical Research Institute, L’Hospitalet de Llobregat, Barcelona, Spain; Infection and Cancer Laboratory, Cancer Epidemiology Research Program, Catalan Institute of Oncology/Bellvitge Biomedical Research Institute, L’Hospitalet de Llobregat, Barcelona, Spain; Centro de investigación Biomédica en Red en Epidemiología y Salud Pública, Ministerio de Ciencia e Innovación en Epidemiología y Salud Pública, Madrid, Spain; Viral and Bacterial Infections Worldwide Program, Barcelona Institute for Global Health, Barcelona, Spain; Pathology Unit, Bellvitge University Hospital/Bellvitge Biomedical Research Institute, L’Hospitalet de Llobregat, Barcelona, Spain; Pathology Unit, Bellvitge University Hospital/Bellvitge Biomedical Research Institute, L’Hospitalet de Llobregat, Barcelona, Spain; Institute of Microbiology and Immunology, Faculty of Medicine, University of Ljubljana, Ljubljana, Slovenia; Infection and Cancer Laboratory, Cancer Epidemiology Research Program, Catalan Institute of Oncology/Bellvitge Biomedical Research Institute, L’Hospitalet de Llobregat, Barcelona, Spain; Centro de investigación Biomédica en Red en Epidemiología y Salud Pública, Ministerio de Ciencia e Innovación en Epidemiología y Salud Pública, Madrid, Spain; HIV and STD Unit, Infectious Diseases Department, Bellvitge University Hospital/Bellvitge Biomedical Research Institute, L’Hospitalet de Llobregat, Barcelona, Spain; Clinical Research Support Area, Department of Clinical Pharmacology, Germans Trias i Pujol University Hospital, Badalona, Spain; HIV and STD Unit, Infectious Diseases Department, Bellvitge University Hospital/Bellvitge Biomedical Research Institute, L’Hospitalet de Llobregat, Barcelona, Spain

**Keywords:** anal cancer, anal dysplasia, HPV, HSIL, men who have sex with men

## Abstract

**Background:**

This study was conducted to evaluate screening procedures for anal high-grade squamous intraepithelial lesions (HSILs) with anal liquid-based cytology (aLBC) and biomarkers to identify candidates for high-resolution anoscopy (HRA).

**Methods:**

This cross-sectional study included men who have sex with men with HIV. Participants underwent HRA, aLBC, and biomarker testing. Three screening procedures were compared with aLBC: biomarker alone, cytology and biomarker in all, and cytology and reflex biomarkers (biomarkers applied if aLBC results were atypical squamous cells of undetermined significance or low-grade squamous intraepithelial lesion). Biomarkers included Linear Array (LA), LA for 14 high-risk human papillomavirus (LA 14 HR-HPV) genotypes, LA HPV-16, Hybrid Capture 2 (HC2), E6/E7 mRNA, and E6/E7 mRNA HPV-16.

**Results:**

Of 354 participants, 179 (50.6%) had atypical squamous cells of undetermined significance or worse, requiring HRA (sensitivity, 80%; specificity, 57.3%; area under the curve, 0.687; reference, biopsy-proven HSIL). Cytology and reflex biomarkers per E6/E7 mRNA, LA 14 HR-HPV, and HC2 and the biomarker-alone procedure with HC2 showed comparable accuracy (sensitivities: 71.6%, 78.8%, 73.1%, 75.7%; specificities: 73.5%, 67.9%, 76.1%, 65.5%; areas under the curve: 0.726, 0.734, 0.746, 0.706) with fewer HRA referrals (number needed to diagnose: 2.2, 2.1, 2, 2.4).

**Conclusions:**

Our findings suggest that E6/E7 mRNA, LA 14 HR-HPV, and HC2 in the cytology and reflex biomarkers procedure, as well as HC2 in the biomarker-alone procedure, can improve anal HSIL screening effectiveness.

Anal squamous cell carcinoma is a rare disease in the general population. Its incidence is significantly higher in certain groups, such as men who have sex with men (MSM) with HIV, in whom incidence can reach 78 to 402 cases per 100 000 person-years [1–4]. The detection and treatment of its precursor lesion, high-grade squamous intraepithelial lesions (HSILs), reduce the incidence of anal cancer [4, 5]. The gold standard for HSIL detection, high-resolution anoscopy (HRA)–guided biopsy, is expensive and requires an experienced team and specialized equipment, making it unavailable in many hospitals [6, 7]. Besides, the invasive nature of HRA, the potential discomfort for patients, and the likelihood of bleeding or other complications can increase the morbidity associated with the diagnostic technique. No consensus has been reached on how to perform anal HSIL screening [8]. The International Anal Neoplasia Society provides a framework to inform evidence-based screening practices, emphasizing the importance of reducing HRA referrals [9]. Anal liquid-based cytology (aLBC) diagnostic operating characteristics correlate imperfectly with histologic biopsy results [10, 11], leading to excess referrals for HRA. Given these challenges, there is increasing interest in identifying biomarkers that could improve screening programs.

Human papillomavirus (HPV) infection is considered a necessary cause for anal squamous cell carcinoma development. The detection of HPV DNA as a biomarker in screening algorithms remains controversial, given the high prevalence of anal infection with high-risk HPV (HR-HPV) genotypes, approximately 80%, among MSM with HIV [12–14]. The HPV oncoproteins E6 and E7 play a pivotal role in the development of HPV-related cancers by initiating a cascade of events that leads to carcinogenic transformation. Therefore, the detection of E6 and E7 could enable the discrimination between transitory and oncogenic HPV infections [15, 16], which may be useful in screening for HSIL to identify candidates for HRA [17–19].

In this study, we assessed the accuracy of various anal dysplasia screening procedures using different combinations of aLBC results, HPV DNA, and/or E6/E7 mRNA testing to identify candidates for HRA.

## METHODS

### Study Design

We conducted a cross-sectional study of outpatients treated at the HIV and STD Unit of Bellvitge University Hospital, Barcelona, Spain. The study was approved by the center's institutional review board (PR 161/16). Written informed consent was obtained from all patients, and the study was conducted in accordance with the Declaration of Helsinki, good clinical practice guidelines, and the Spanish regulatory requirements. Confidentiality was guaranteed according to current Spanish legislation (LOPD 3/2018). This article complies with the STARD guidelines.

### Study Population and Period

The study population comprised participants from the ELAVI cohort, a prospective study aimed at evaluating the accuracy of viral biomarkers for detecting HSIL incidence, which included all MSM with HIV (age ≥18 years) eligible for anal dysplasia screening. At the same visit, each participant underwent a digital anal rectal and perianal examination, an anal smear for aLBC and detection of viral biomarkers, and HRA.

### aLBC Samples and Cytologic Diagnosis

aLBC sampling was performed with a Dacron Collection Swab (Deltalab), which was inserted into the anal canal and rotated for 40 seconds before being placed into a ThinPrep Pap test vial containing PreservCyt liquid-based medium (Hologic). Thin-layer slides were stained per the Papanicolaou method.

Cytologic diagnosis was based on the Bethesda system [20]: negative for intraepithelial lesion and malignancy (NILM), atypical squamous cells of undetermined significance (ASC-US), low-grade squamous intraepithelial lesion (LSIL), HSIL, atypical squamous cells that cannot exclude HSIL (ASC-H), and anal cancer. The diagnostic cytology workup was performed by the same 2 pathologists. If the sample did not contain enough cells to confirm a cytologic diagnosis, the result was reported as inadequate.

### HRA and Histologic Diagnosis

All participants underwent HRA at the same visit as aLBC. An anoscope was inserted into the anal canal, and 3%–5% acetic acid was used for staining. The anal canal was visualized through the video colposcope, and biopsies of suspicious lesions were taken with a mini-forceps and placed in formalin solution. Histology results were based on the LAST project (Lower Anogenital Squamous Terminology) [21, 22]: negative, LSIL, HSIL, or anal cancer. If multiple areas were biopsied, the highest grade of histologic abnormality was used. In cases where no biopsy was taken, participants were considered to have a negative histologic result, as biopsies were not performed randomly. The diagnostic histology workup was performed by the same pathologist. The pathologists reported cytology and histology findings independently and without knowledge of concurrent or previous results.

### Detection of HPV Biomarkers

The Linear Array HPV Genotyping Test (LA; Roche Molecular Systems) is a polymerase chain reaction–based qualitative test that identifies 37 HR-HPV and low-risk HPV genotypes and uses the biotinylated primers sets PGM09/PGMY11 and PC04/GH20 for amplification of the HPV *L1* gene and the human beta-globin gene. Since LA is unable to identify HPV-52 alone in samples containing HPV-33, HPV-35, and/or HPV-58, HPV-52 type–specific real-time polymerase chain reaction was performed to confirm HPV-52 infections when needed [23]. For this study we conducted an analysis of all 37 HPV genotypes (LA), focusing on the 14 HR-HPV genotypes present in the E6/E7 mRNA test (LA 14 HR-HPV) and on detection of HPV-16 (LA HPV-16).

The Hybrid Capture 2 HPV DNA Test (HC2; Qiagen) is a validated nucleic acid hybridization assay with signal amplification that enables qualitative detection of the DNA of 13 HR-HPV genotypes (HPV-16, 18, 31, 33, 35, 39, 45, 51, 52, 56, 58, 59, and 68).

The Aptima HPV Test (E6/E7 mRNA; Gen-Probe) is a qualitative nucleic acid amplification test for detection of E6/E7 mRNA from 14 HR-HPV genotypes (HPV-16, 18, 31, 33, 35, 39, 45, 51, 52, 56, 58, 59, 66, and 68). All positive samples were genotyped by the Aptima HPV 16 18/45 Genotype Assay, which detected E6/E7 mRNA from HPV-16 (E6/E7 HPV-16) and from HPV-18 and HPV-45 together.

### DNA Extraction

Total nucleic acids were extracted from 5 mL of the aLBC sample with the Maxwell 16 LEV Blood DNA Kit (Promega Corp). DNA was eluted with 100 μL of nuclease-free water.

### Screening Procedures

#### Anal Liquid-Based Cytology

**Figure 1.**
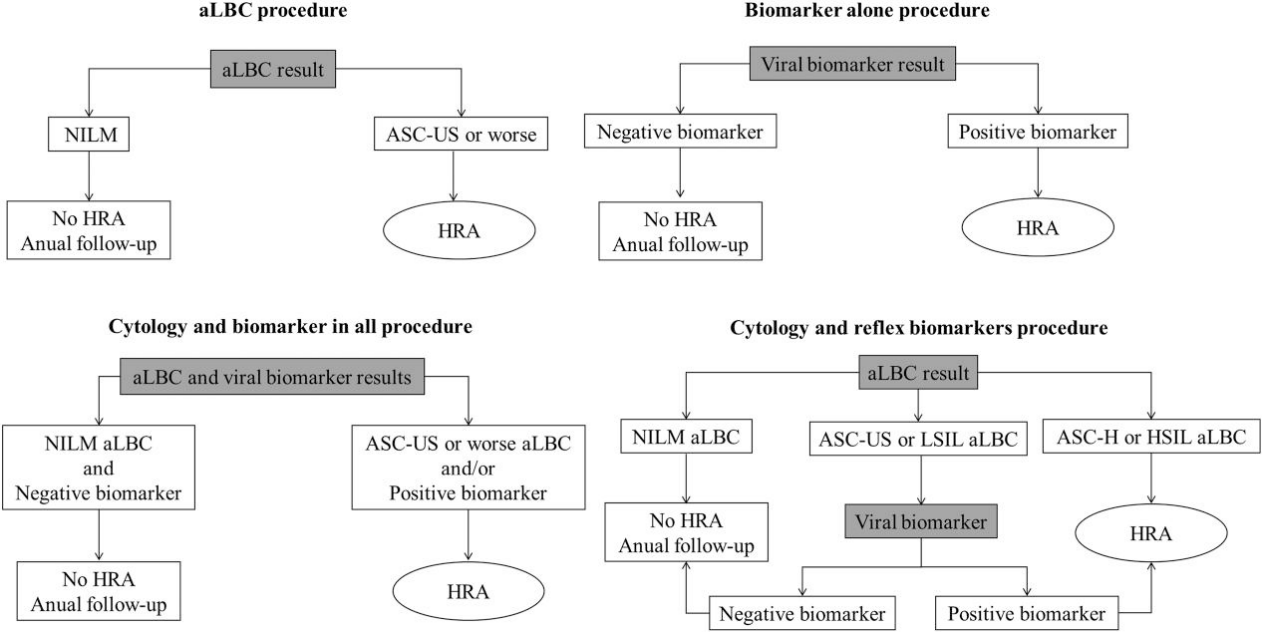
Screening procedures. aLBC, anal liquid-based cytology; ASC-H, atypical squamous cells that cannot exclude HSIL; ASC-US, atypical squamous cells of undetermined significance; HRA, high-resolution anoscopy; HSIL, high-grade squamous intraepithelial lesion; LSIL, low-grade squamous intraepithelial lesion; NILM, negative for intraepithelial lesion and malignancy.

Currently, the most common screening strategy is based on aLBC: if the result is ASC-US or worse, the patient is referred for HRA. We evaluated 3 procedures, each of which included 6 biomarkers (Figure 1): LA, LA 14 HR-HPV, LA HPV-16, HC2, E6/E7 mRNA, and E6/E7 mRNA HPV-16.

#### Biomarker

If the result of the biomarker test was positive, the patient was referred for HRA.

#### Cytology and Biomarker in All

Simultaneous aLBC and biomarker testing were the first step. If the aLBC result was ASC-US or worse and/or the biomarker test result was positive, the patient was referred for HRA.

#### Cytology and Reflex Biomarkers

aLBC was the first step. If the result was ASC-H or HSIL, the patient was referred to HRA. If the result was ASC-US or LSIL, a viral biomarker test was performed, and if the result was positive, the patient was referred for HRA.

### Statistical Analyses

The sample size was calculated for the ELAVI project, which aims to evaluate the accuracy of viral biomarkers for detecting HSIL incidence over 2 to 4 years. Based on a 50% prevalence of E6/E7 mRNA in MSM with HIV and HPV, with an annual HSIL incidence of 8.5% to 15.4%, it was expected that 17% would develop HSIL by 24 months, with a 15% higher incidence in E6/E7 expressers. To achieve 80% power and a 5% type I error rate, 355 participants were needed to ensure 270 for analysis, accounting for a 5% dropout rate.

Baseline characteristics were summarized by standard descriptive statistics. Continuous variables were expressed as mean and SD. Categorical variables were described as the number of cases and percentage of the total. Prior to the statistical analysis, the normality of distributions for continuous variables was tested. Quantitative variables were compared with the *t* test or Mann-Whitney test; qualitative variables were compared with the χ^2^ or Fisher exact test. Statistical significance was set at *P* < .05. The sensitivity, specificity, positive predictive value (PPV), negative predictive value (NPV), and area under the curve (AUC) for each screening procedure with each viral biomarker were calculated and compared with the aLBC procedure. The number needed to diagnose (NND) was used to quantify diagnostic performance, reflecting how many individuals need to be tested to correctly diagnose 1 case based on the test's sensitivity and specificity, NND = 1 / (sensitivity + specificity – 1), and defining referrals to HRA. Statistical analyses were carried out with R version 4.1.2 for Windows.

## RESULTS

### Characteristics of Participants

Between June 2016 and December 2018, 354 participants were included in the cohort. The mean age was 45.3 years (SD, 11.2), and the mean CD4 T-cell count was 802 cells/µL (SD, 333). Furthermore, 87.3% of the participants had undetectable plasma HIV-1 RNA (Table 1). After comparing the characteristics of participants with and without biopsy-proven HSIL, we found differences only in the number of sexual partners during the previous 6 months (mean [SD], 2.84 [6.32] and 5.20 [12.4], respectively; *P* = .031; Supplementary Table 1).

**Table 1.** Baseline Characteristics of the 354 Participants

	Mean ± SD or No. (%)	Total or Subtotal
Sociodemographic characteristic		
Age, y	45.3 ± 11.2	354
Current smokers	140 (39.5)	354
Ex-smokers	62 (17.5)	354
HIV-related variables		
Months since diagnosis	126 ± 91.5	354
Nadir CD4 T-cell count		
Cells/µL	335 ± 259	354
<200 cells/µL	109 (30.8)	354
Current CD4 T-cell count		
Cells/µL	802 ± 333	354
<200 cells/µL	5 (1.41)	354
Undetectable viral load	309 (87.3)	354
Sexual behavior		
Age at first sexual intercourse, y	17.8 ± 3.87	333
Lifetime sexual partners		336
<30	119 (35.4)	
30–50	82 (24.4)	
51–100	48 (14.3)	
>100	87 (25.9)	
Use of condoms		337
Always	116 (34.4)	
Almost always	148 (43.9)	
Sometimes	61 (18.1)	
Never	12 (3.6)	
Transactional sex	31 (9.2)	336
Receptive anal intercourse	254 (74.9)	339
No. of sexual partners during the previous 6 mo	4.69 ± 11.4	321
History of anogenital warts	136 (38.4)	354
ChemSex during the previous 6 mo	66 (30)	220
GHB, mephedrone, and/or methamphetamine	19 (28.8)	66
Slamming during the previous 6 mo	3 (1.5)	196

Abbreviation: GHB, gamma hydroxybutyrate.

### Cytology and Histology Results

The cytology results were as follows: 49.4% (175/354) NILM, 16.4% (58/354) ASC-US, 15.8% (56/354) LSIL, 13% (46/354) ASC-H, 2.8% (10/354) HSIL, and 2.5% (9/354) inadequate. Biopsies were taken in 76.8% (272/354) of participants, with 30.8% (109/354) NILM, 24.9% (88/354) LSIL, and 21.2% (75/354) HSIL (Table 2).

**Table 2.** Anal Liquid-Based Cytology and Histology Results

	HRA, No. (%)				
ALBC	No Biopsy	Negative	LSIL	HSIL	Total
NILM	52	72	36	15	175 (49.4)
ASC-US	18	18	9	13	58 (16.4)
LSIL	6	5	23	22	56 (15.8)
ASC-H	2	7	13	24	46 (13)
HSIL	2	4	3	1	10 (2.8)
Inadequate	2	3	4	0	9 (2.5)
Total	82 (23.2)	109 (30.8)	88 (24.9)	75 (21.2)	354 (100)

Abbreviations: ALBC, anal liquid-based cytology; ASC-H, atypical squamous cells that cannot exclude HSIL; ASC-US, atypical squamous cells of undetermined significance; HRA, high-resolution anoscopy; HSIL, high-grade squamous intraepithelial lesion; LSIL, low-grade squamous intraepithelial lesion; NILM, negative for intraepithelial lesion and malignancy.

### Detection of Viral Biomarkers

LA was positive in 91.5% (324/354) of participants and LA 14 HR-HPV in 71.2% (252/354). HPV-16 was the most frequently detected genotype (23.4%, 82/354; Supplementary Table 2 for genotypes). According to LA, the prevalence of a multiple-genotype infection was 69.8% (247/354) and that of multiple HR genotype, 46.5% (163/354). Per HC2, HPV-DNA was detected in 42.7% (151/354) of participants. E6/E7 mRNA was detected in 49.7% (176/354), with positive E6/E7 HPV-16 results in 16.1% (57/354). All biomarkers were more frequently positive in participants with biopsy-proven HSIL than in those without (Supplementary Table 1).

### Accuracy of the Screening Procedures

The sensitivity of the aLBC procedure was 80%, with a specificity of 57.3%, PPV 33.5%, and NPV 91.4%. The AUC was 0.687. Table 3 presents the sensitivity, specificity, PPV, NPV, and AUC of the 3 procedures, each with 6 biomarkers. The “cytology and biomarker in all” procedure had the highest sensitivity for all biomarkers but the lowest specificity and PPV. The cytology and reflex biomarkers procedure presented lower sensitivity but better specificity and PPV when compared with the “cytology and biomarker in all” procedure and showed the highest AUC.

**Table 3.** Sensitivity, Specificity, PPV, NPV, and AUC According to HPV Biomarkers Used in the 3 Screening Procedures

	Biomarker Alone					Cytology and Biomarker in All					Cytology Reflex Biomarkers				
	Se	Sp	PPV	NPV	AUC	Se	Sp	PPV	NPV	AUC	Se	Sp	PPV	NPV	AUC
aLBC	80	57.3	33.5	91.4	0.687	…	…	…	…	…	…	…	…	…	…
LA	91.4	27.5	23.7	92.9	0.594^a^	95.7	20.4	22.9	95.1	0.581^a^	80.6	64	36.2	92.2	0.723
LA 14 HR-HPV	94	34.3	25.5	96	0.642	98.6	24.6	24.1	98.6	0.616^a^	78.8	67.9	38.2	92.7	0.734
LA HPV-16	47.1	82.7	40.2	86.4	0.649	87.1	49.6	29.9	94	0.684	58.2	84.5	48.8	88.8	0.713
HC2	75.7	65.5	35.1	91.6	0.706	88.6	44.4	28.2	94	0.665	73.1	76.1	43.8	91.8	0.746
E6/E7 mRNA	78.6	57.4	31.2	91.6	0.680	92.9	39.1	28.2	94	0.660	71.6	73.5	43.8	91.8	0.726
E6/E7 mRNA HPV-16	38.6	89.4	47.4	85.5	0.640	85.7	52.8	30.9	93.8	0.693	50.7	86.4	48.6	87.4	0.686

Biopsy-proven high-grade squamous intraepithelial lesion was used as reference test. All data are presented as a percentage except AUC.

Abbreviations: aLBC, anal liquid-based cytology; AUC, area under the curve; E6/E7 mRNA, E6/E7 mRNA detection for all 14 high-risk genotypes in the test; E6/E7 mRNA HPV-16, E6/E7 mRNA detection for HPV-16; HC2, Hybrid Capture 2 HPV DNA test; HPV, human papillomavirus; LA, Linear Array HPV DNA test for all 37 genotypes in the test; LA 14 HR-HPV, Linear Array HPV DNA test for the 14 most important high-risk genotypes; LA HPV-16, Linear Array HPV DNA test for HPV-16; NPV, negative predictive value; PPV, positive predictive value; Se, Sensitivity; Sp, Specificity.

^a^ *P* < .05 vs aLBC.

### Referral for HRA With Each Screening Procedure

Table 4 presents the number of biopsy-proven HSILs and the NND for each of the 18 screening strategies (3 procedures, each with 6 biomarkers). The biopsy-proven HSIL detection rate for aLBC was 80%, with an NND of 2.7. Strategies with a lower NND than aLBC and a comparable biopsy-proven HSIL detection rate were HC2 in the biomarker-alone procedure and LA, LA 14 HR-HPV, HC2, and E6/E7 mRNA in the cytology and reflex biomarkers procedure.

**Table 4.** Participants Referred for HRA, Biopsy-Proven HSILs, and NND vs aLBC of the 18 Screening Strategies

	Biomarker Alone		Cytology and Biomarker in All		Cytology and Reflex Biomarkers	
	Biopsy-Proven HSIL Detection (n = 75)	NND	Biopsy-Proven HSIL Detection (n = 75)	NND	Biopsy-Proven HSIL Detection (n = 75)	NND
aLBC	60	2.7	…	…	…	…
LA	73 (97.3)^a^	5.3	74 (98.7)^a^	6.21	59 (78.7)	2.2
LA 14 HR-HPV	68 (90.7)	3.5	73 (97.3)^a^	4.31	57 (76)	2.1
LA HPV-16	34 (57.3)	3.4	63 (84)	2.7	40 (53.3)	2.3
HC2	58 (77.3)	2.4	67 (89.3)	3	54 (72)	2
E6/E7 mRNA	62 (82.7)	2.9	70 (93.3)^a^	3.1	55 (73.3)	2.2
E6/E7 mRNA HPV-16	31 (41.3)	3.6	62 (82.6)	2.6	38 (50.7)	2.7

Data are presented as No. (%). Only *P* values showing significant differences in favor of the screening strategy vs aLBC are notated; otherwise, differences are nonsignificant.

Abbreviations: aLBC, anal liquid-based cytology; E6/E7 mRNA, E6/E7 mRNA detection for all 14 high-risk genotypes in the test; E6/E7 mRNA HPV-16, E6/E7 mRNA detection for HPV-16; HC2, Hybrid Capture 2 HPV DNA test; HRA, high-resolution anoscopy; HSIL, high-grade squamous intraepithelial lesion; LA, Linear Array HPV DNA test for all 37 genotypes in the test; LA 14 HR-HPV, Linear Array HPV DNA test for the 14 most important high-risk genotypes; LA HPV-16, Linear Array HPV DNA test for HPV-16; NND, number needed to diagnose.

^a^ *P* < .001.

## DISCUSSION

In this study, we evaluated 3 procedures for anal dysplasia screening, each with 6 biomarkers based on HPV DNA and E6/E7 mRNA detection in MSM with HIV, and we compared them with the standard-of-care aLBC procedure.

The main results indicate that the cytology and reflex biomarkers procedure per LA 14 HR-HPV, HC2, or E6/E7 mRNA and the biomarker-alone procedure with HC2 can improve the effectiveness of anal dysplasia screening programs. These strategies demonstrated accuracy comparable to the aLBC procedure, with NND values indicating fewer HRA referrals, highlighting their greater efficiency, along with an adequate biopsy-proven HSIL detection rate.

The ANCHOR study demonstrated that treating anal HSIL significantly decreases the incidence of anal cancer among people with HIV [4]. These findings provide support for implementing anal dysplasia screening programs and emphasize the importance of conducting further research to improve their effectiveness [4].

HRA presents several challenges in routine clinical practice. It is often unpopular among patients and can result in noncompliance and consequent loss to follow-up [24]. Additionally, HRA requires an experienced team and specialized equipment, thus preventing its use in many hospitals [6, 7]. Excess referral for HRA without HSIL diagnosis can lead to increased health care delivery costs, rendering large-scale anal dysplasia screening programs unfeasible [6, 7]. Therefore, it is imperative to develop screening strategies that reduce referral for HRA.

In our study, the accuracy of the aLBC procedure was consistent with that reported in other studies [10, 25]. We devised 18 screening strategies using 3 screening procedures and 6 viral biomarkers. The biomarker procedure used only biomarkers to assess referral for HRA. The 2 other procedures combined aLBC and biomarkers. In the “cytology and biomarker in all” procedure, all participants underwent biomarker testing and aLBC, whereas in the cytology and reflex biomarkers procedure, biomarkers were assessed only if aLBC revealed ASC-US or LSIL. For the biomarker procedure, the sensitivity of anal HPV DNA testing was high; however, its specificity was low among MSM with HIV due to the high prevalence of anal HPV infection [12–14].

With LA, the overall prevalence of anal HPV infection was 91.5%. When analysis was restricted to LA 14 HR-HPV, prevalence decreased to 71.2%, which was still high. Accuracy results were consistent with those reported in the meta-analysis by Clarke et al [10]. LA and LA 14 HR-HPV in the biomarker procedure did not improve accuracy when compared with the aLBC procedure, and the NND was higher.

When HC2 was used in the biomarker procedure, no significant differences in the AUC were found when compared with the aLBC procedure. Studies based on HC2 [26] show higher sensitivity and lower specificity than the current study. However, these differences could be due to the techniques applied, since Salit et al used ThinPrep samples whereas we used extracted DNA [26]. In our study, HC2 reduced referral for HRA, showing a lower NND while maintaining a comparable biopsy-proven HSIL rate. In the SPANC study [27], the rate of progression from HSIL to anal cancer was low (0.324 per 100 person-years). Considering this slow progression and the fact that screening is conducted annually, a screening method that detects at least 70% of biopsy-proven HSIL appears to be sufficient. In clinical practice, HPV DNA tests such as HC2 are being used less over time due to their lack of genotyping and significant cross-reactivity with low-risk HPV genotypes, along with a “gray zone” in result interpretation. Yet, HC2 remains well established in HPV detection, offering comparability with past studies. Additionally, HC2 is still widely used in many health care systems due to cost and infrastructure issues, ensuring the generalizability of results across different clinical environments.

The accuracy of E6/E7 mRNA tests was comparable to the findings in the meta-analysis by Clarke et al [10]. When compared with aLBC, the accuracy of E6/E7 mRNA and the detection rate of biopsy-proven HSIL were similar, but the NND was slightly higher.

For all the biomarkers evaluated, the “cytology and biomarker in all” procedure yielded higher sensitivity than either the biomarker procedure or the aLBC procedure, resulting in higher biopsy-proven HSIL detection rates. However, this procedure had lower specificity leading to more HRA referrals, as indicated by NND values higher than those of aLBC. A further disadvantage is the need to perform the biomarker test and aLBC test on all patients. Therefore, this procedure does not appear to have clinical relevance if our goal is to reduce HRA referrals and minimize costs. Kimura et al also observed this increased HRA referral rate when using a similar procedure that combined cytology with biomarkers, with HPV DNA detection as the biomarker [28].

Finally, the cytology and reflex biomarkers procedure resulted in higher specificity than the other procedures or aLBC procedure in most cases. Viciana et al [25] developed a comparable screening strategy using HC2 only if aLBC resulted in LSIL. The authors concluded that patients with LSIL and no detection of HR-HPV may not require HRA. Other publications have suggested using reflex HR-HPV detection in cases of aLBC results of ASC-US [28, 29]. In our study, the cytology and reflex biomarkers represent refinement of these strategies by evaluating biomarkers in cases where aLBC revealed ASC-US or LSIL, while broadening the range of viral biomarkers studied.

With this procedure, by using E6/E6 mRNA, LA 14 HR-HPV, and HC2, HRA referrals were lower than with the aLBC procedure, with NND values of 2.2, 2, and 2.1, respectively, while maintaining biopsy-proven HSIL detection rates >70% and an AUC comparable to that of aLBC. Another advantage is the remarkable saving of resources in the cytology and reflex biomarkers, since biomarkers must be assessed only if aLBC results in LSIL or ASC-US (32.2% of participants).

One strength of our study is that the proportion of inadequate cytologic sample results was lower than reported elsewhere, reinforcing the validity of our findings [10]. Another remarkable strength is the complete availability of results for all biomarkers for each participant, as well as the simultaneous performance of aLBC and HRA at the same visit, thus reducing the risk of measurement bias.

A potential limitation of the study is that the performance of aLBC and HRA during a single visit necessitates the use of a nonirritative anal smear collection device, which may be less precise than other devices, such as the brushes more commonly used in clinical settings where aLBC is performed [30]. Another limitation of the study is that LA is no longer commercially available. However, its use did not reduce referrals for HRA in any procedure, except with LA 14 HR-HPV in cytology and reflex biomarkers. Despite its limited use in clinical practice, LA remains valuable for epidemiologic and research purposes. It also played a key role in validating newer HPV tests, such as Cobas 4800, with no observed difference in sensitivity or specificity [31]. Additionally, current clinical management algorithms are often based on data from studies using LA, reinforcing its impact on modern guidelines.

In conclusion, E6/E7 mRNA, LA 14 HR-HPV, and HC2 in the cytology and reflex biomarkers procedure, as well as HC2 in the biomarker-alone procedure, appear to be the best strategies for identifying candidates for HRA, as they demonstrate accuracy comparable to that of the aLBC procedure while reducing HRA referrals without compromising the biopsy-proven HSIL detection rate. These findings suggest that these strategies have the potential to enhance the effectiveness of anal dysplasia screening programs.

Future prospective longitudinal and cost-effectiveness studies are needed to validate these findings.

## Supplementary Material

ofae735_Supplementary_Data

## Supplementary Data

Supplementary materials are available at *Open Forum Infectious Diseases* online. Consisting of data provided by the authors to benefit the reader, the posted materials are not copyedited and are the sole responsibility of the authors, so questions or comments should be addressed to the corresponding author.

## Notes

***Acknowledgments.*** This article is dedicated to our late colleague Dr Elena Ferrer, who made significant contributions to the implementation of our anal dysplasia screening program and to the study design.

Investigators from the HIV and STD Unit, in collaboration with L. T., recruited participants, performed anal smears and HRA, and designed the screening procedures. Investigators from the Infection and Cancer Laboratory prepared and analyzed biomarkers, supporting study design and development. Investigators from the Pathology Unit provided the cytologic and histologic diagnoses. M. P. provided the Linear Array kits, thus enabling biomarker testing for all participants. S. V. and S. D. S. provided support in designing the study and drafting the article.

We thank CERCA Programme/Generalitat de Catalunya for institutional support.

All coauthors reviewed this article.

***Financial support*.** This work was supported by the Instituto de Salud Carlos III through the project CIBERESP (Consorcio de Investigación Biomédica en Red de España; CB06/02/0073), a Gilead Science grant (GLD17/00285), and the project FIS (Fondo de Investigación Sanitaria; PI16/01056), which were used to acquire the viral biomarkers. This work was also supported by the Secretariat for Universities and Research of the Ministry of Business and Knowledge of the Government of Catalonia (2021SGR01354).
