# Supplementary material for: Using Anal Cytology and Human Papillomavirus DNA and E6/E7 mRNA Detection to Optimize High-Resolution Anoscopy Referrals in Men Who Have Sex With Men With HIV
Source: Open Forum Infect Dis. 2024 Dec 27;12(1):ofae735. doi: 10.1093/ofid/ofae735 (PMC11770276; doi:10.1093/ofid/ofae735)
Supplement: Supplementary file 1 [file ofae735_supplementary_data.zip › Footnote page.odt]

Conflict of interest statement: The Cancer Epidemiology Research Program, Catalan Institute of Oncology (ICO-IDIBELL) has received sponsorship for grants from Merck & Co., Roche, GSK, IDT, Hologic, and Seegene, that are not related to this study. Hologic generously provided the reagents for Aptima Assay and conducted the testing at no cost to our research project but did not play any role in the study design, conduction, data analysis; in the writing of the report; and in the decision of submitting the article for publication. The other authors declare no conflicts of interest.

Funding statement: This study was supported by Instituto de Salud Carlos III through the projects CIBERESP “Consorcio de Investigación Biomédica en Red de España” [CB06/02/0073], a Gilead Science grant [GLD17/00285], and FIS “Fondo de Investigación Sanitaria” [PI16/01056], which were used to acquire the viral biomarkers. This work was also supported by the Secretariat for Universities and Research of the Ministry of Business and Knowledge of the Government of Catalonia [2021SGR01354].
