# Supplementary material for: Using Anal Cytology and Human Papillomavirus DNA and E6/E7 mRNA Detection to Optimize High-Resolution Anoscopy Referrals in Men Who Have Sex With Men With HIV
Source: Open Forum Infect Dis. 2024 Dec 27;12(1):ofae735. doi: 10.1093/ofid/ofae735 (PMC11770276; doi:10.1093/ofid/ofae735)
Supplement: Supplementary file 1 [file ofae735_supplementary_data.zip › Supplementary Table 1.odt]

Supplementary table 1: Comparison of baseline characteristics and HPV biomarkers between participants with biopsy-proven HSIL and participants without biopsy-proven HSIL.

|  | Biopsy-proven HSIL (n=75) | Non biopsy-proven HSIL (n=279) | p-value | N |
| --- | --- | --- | --- | --- |
| Sociodemographic characteristics |  |  |
| Age (years), mean (SD) | 44·1 (9·8) | 45·6 (11·6) | 0·271 | 354 |
| Current smokers, n (%) | 33 (44%) | 119 (42·7%) | 0·978 | 354 |
| HIV related variables |  |  |
| Months since diagnosis, mean (SD) | 116 (92·2) | 128 (91·4) | 0·311 | 354 |
| Nadir CD4 T-cell count <200 cells/µL, N (%) | 18 (24%) | 91 (32·6%) | 0·196 | 354 |
| Current CD4 T-cell count <200 cells/µL, N (%) | 3 (4%) | 2 (0·72%) | 0·066 | 354 |
| Undetectable viral load, N (%) | 66 (88%) | 243 (87·1%) | 0·989 | 354 |
| Sexual behavior |  |  |
| Age at first sexual intercourse, mean (SD) | 17·4 (3·08) | 17·8 (4·06) | 0·324 | 333 |
| Lifetime sexual partners, N (%): |  |  | 0·585 | 335 |
| <30 | 26 (35·6%) | 93 (35·4%) |  |  |
| 30-50 | 19 (26%) | 63 (24%) |  |  |
| 50-100 | 13 (17·8%) | 35 (13·3%) |  |  |
| >100 | 15 (20·5%) | 72 (27·4%) |  |  |
| Use of condoms, N (%): |  |  | 0·533 | 337 |
| Always | 27 (37·5%) | 89 (33·6%) |  |  |
| Almost always | 33 (45·8%) | 115 (43·4%) |  |  |
| Sometimes | 9 (12·5%) | 52 (19·6%) |  |  |
| Never | 3 (4·17% | 9 (3·4%) |  |  |
| Receptive anal intercourse, N (%) | 51 (69·9%) | 203 (76·3%) | 0·330 | 339 |
| Number of sexual partners during the previous 6 months, mean, (SD) | 2·8 (6.3) | 5·2 (12·4) | 0·031 | 321 |
| History of anogenital warts, N (%) | 32 (42·7%) | 104 (37·3%) | 0·473 | 354 |
| ChemSex during the previous 6 months, N (%) | 5 (9·4%) | 16 (9·6%) | 1 | 220 |
| Viral biomarkers |  |
| LA | 72 (96%) | 243 (87·1%) | <0·001 | 354 |
| LA 14 HR-HPV | 68 (90·6%) | 179 (64·2%) | <0·001 | 354 |
| LA HPV 16 | 34 (45·3%) | 48 (17·2%) | 0·02 | 354 |
| HC2 | 58 (77·3%) | 93 (33·3%) | <0·001 | 354 |
| E6/E7 mRNA-test | 62 (82·7%) | 114 (40·9%) | <0·001 | 354 |
| E6/E7 mRNA HPV 16 | 31 (44%) | 40 (14·3%) | <0·001 | 354 |

HSIL: high -grade squamous intraepithelial lesion LA: Linear Array HPV DNA test for all the 37 genotypes included in the test; LA 14 HR-HPV: Linear Array HPV DNA test for the 14 most important high-risk genotypes; LA HPV-16: Linear Array HPV DNA test for HPV-16; HC2: Hybrid Capture 2 HPV DNA test; E6/E7 mRNA-test: E6/E7 mRNA detection for all the 14 high-risk genotypes included in the test; E6/E7 mRNA HPV 16: E6/E7 mRNA detection for HPV-16.
