# Supplementary material for: Using Anal Cytology and Human Papillomavirus DNA and E6/E7 mRNA Detection to Optimize High-Resolution Anoscopy Referrals in Men Who Have Sex With Men With HIV
Source: Open Forum Infect Dis. 2024 Dec 27;12(1):ofae735. doi: 10.1093/ofid/ofae735 (PMC11770276; doi:10.1093/ofid/ofae735)
Supplement: Supplementary file 1 [file ofae735_supplementary_data.zip › Supplementary Table 2.odt]

Supplementary table 2: Results for the 37 HPV genotypes analyzed.

| 14 HR-HPV genotypes |
| --- |
| 16 | 82 (23·4%) |
| 52 | 60 (16·9%) |
| 31 | 54 (15·3%) |
| 58 | 54 (15·3%) |
| 51 | 48 (13·6%) |
| 59 | 47 (13·3%) |
| 66 | 43 (12·1%) |
| 45 | 37 (10·5%) |
| 33 | 35 (10%) |
| 68 | 32 (9·1%) |
| 35 | 31 (8·8%) |
| 39 | 30 (8·5%) |
| 18 | 26 (7·4%) |
| 56 | 19 (5·4%) |
| Other HPV genotypes |
| 53 | 91 (25·7%) |
| 84 | 60 (16·9%) |
| IS39 | 6 (1·7%) |
| CP6108 | 57 (16·1%) |
| 61 | 56 (15·8%) |
| 55 | 55 (15·5%) |
| 70 | 54 (15·3%) |
| 62 | 53 (15%) |
| 6 | 47 (13·3%) |
| 54 | 46 (13%) |
| 81 | 43 (12·1%) |
| 11 | 42 (12%) |
| 72 | 37 (10·5%) |
| 73 | 34 (9·7%) |
| 42 | 31 (8·8%) |
| 83 | 26 (7·4%) |
| 71 | 15 (4·2%) |
| 26 | 12 (3·4%) |
| 82 | 12 (3·4%) |
| 40 | 11 (3·1%) |
| 67 | 11 (3·1%) |
| 69 | 10 (2·8%) |
| 64 | 1 (0·3%) |

HR-HPV: high-risk HPV genotypes
